# Arbuscular mycorrhizal fungi improve the growth and performance in the seedlings of *Leymus chinensis* under alkali and drought stresses

**DOI:** 10.7717/peerj.12890

**Published:** 2022-02-03

**Authors:** Yingnan Wang, Jixiang Lin, Fan Yang, Shuang Tao, Xiufeng Yan, Zhiqiang Zhou, Yuhong Zhang

**Affiliations:** 1 Key Laboratory of Forest Plant Ecology, Ministry of Education, Northeast Forestry University, Harbin, China; 2 College of Landscape Architecture, Northeast Forest University, Harbin, Heilongjiang, China; 3 Zhejiang Provincial Key Laboratory for Water Environment and Marine Biological Resources Protection, College of Life and Environmental Science, Wenzhou University, Wenzhou, China

**Keywords:** Alkali stress, Arbuscular mycorrhizal fungi, Drought stress, *Leymus chinensis*, Interactive effect

## Abstract

Alkali and drought stresses are increasing severe environmental problems throughout the world, especially in the Songnen grassland of northern China. *Leymus chinensis* is the dominant grass species in the Songnen grassland of northern China and the most promising species for grassland restoration. Arbuscular mycorrhizal fungi (AMF) can colonize 80% of vascular plants, which can enhance the growth of host plants and provide extrinsic protection against abiotic stresses. However, little is known about the interaction effect of alkali and drought stresses on plant-AM symbionts. Here, seedlings of *Leymus chinensis* inoculated with or without mycorrhizae were cultivated in soil with 0, 100 or 200 mM NaHCO_3_ under 0, 5 or 10% (w/v) PEG treatment, and the changes in growth, osmotic adjustment substances and ions were measured. The results showed that the interaction of alkali and drought stresses caused greater seedling growth inhibition than either single alkali or drought stress due to ion toxicity and oxidative damage. Mycorrhizae could alleviate the growth inhibition of seedlings under alkali or drought stress. The interaction of alkali and drought stresses did not affect the alleviating effect of mycorrhizae on seedling growth but improved the osmotic regulation ability and ionic balance of the seedlings. Our results clearly show different effects of the interaction of alkali and drought stresses versus a single stress (alkali or drought) on plant development and provide new insights into the positive effect of arbuscular mycorrhizal fungi on host plants under such stress conditions.

## Introduction

Climate warming has already led to frequent and extreme droughts throughout the world and is receiving increasing attention from the global community (Zhang et al., 2018). Northern China contains typical arid and semiarid areas. Since 1960, the frequency and degree of drought in this area have gradually increased. Soil salinization is another environmental problem in this region, where nearly 70% of natural grasslands have been seriously degraded due to soil salinization, which is still increasing in the Songnen grassland of northern China (Lin et al., 2014; Jia et al., 2018). In natural ecosystems, plants are not subject to either salt or drought stress alone but to the interaction of salt and drought stresses (Liu et al., 2014). Previous studies have reported that the inhibitory effect of salt-drought stress is much stronger than that of a single stress (salt or drought) (Perez-Perez et al., 2007). Liu et al. (2014) found that the interaction of salt and drought stresses had a stronger inhibitory effect on the growth of *Tamarix chinensis*. Similar results were found in *Triticum aestivum* (Paul et al., 2019), *Elaeagnus angustifolia* (Chen et al., 2019) and *Populus pruinosa* (Wang et al., 2018). Another remarkable phenomenon observed in Northeast China is soil alkalization (Lin et al., 2017). In addition to osmotic stress and ion injury, the distinctly high pH associated with alkali stress has been demonstrated to be more damaging than salt stress (Wang et al., 2018). However, to the best of our knowledge, most studies have focused only on the interactive effect of drought and salt stresses on plants, with little attention paid to alkali-drought stress interactions.

Arbuscular mycorrhizal fungi (AMF) can colonize most terrestrial plants and thereby enhance the growth of host plants and provide external protection in response to abiotic stresses, such as drought, salt and temperature stresses (Amiri, Nikbakht & Etemadi, 2015; Lin et al., 2017; Zhang et al., 2020). Previous studies have suggested the underlying mechanisms by which AMF improve tolerance to single alkali or drought stress in host plants, including the promotion of water and nutrient uptake (Kumar et al., 2015), improvement of soil aggregate stability (Quiroga et al., 2018), and enhancement of osmotic adjustment (Wang et al., 2019a; Wang et al., 2019b; Wang et al., 2019c; Yang et al., 2020a; Yang et al., 2020b). However, the interactive effect of alkali and drought stresses on the relationship between host plants and AMF is still poorly understood.

*Leymus chinensis*, belonging to the Poaceae family, is a dominant perennial rhizomatous grass. Due to its high vegetative productivity, protein content and palatability, this species is cultivated as a major grass forage product in the Songnen grassland of northern China. Since it develops strong rhizomes and adapts well to saline, alkaline, and drought conditions, this species plays an important role in the establishment and renewal of artificial grasslands and the protection of environments (Ao et al., 2019; Lin et al., 2017; Yang et al., 2020a; Yang et al., 2020b). Hence, understanding the positive effect of AMF on this species is crucial for its use in grassland rehabilitation and restoration. Our previous studies showed a good symbiotic relationship between AMF and this species under salt and alkali stresses (Lin et al., 2017; Wang et al., 2019a; Wang et al., 2019b Wang et al., 2019c; Yang et al., 2020a; Yang et al., 2020b). In the present study, we hypothesized (1) that AMF play an important role in alleviating the inhibitory effect on seedling growth under alkali or drought stress conditions and (2) that the interaction of alkali and drought stresses affects the alleviating effect of AMF in *Leymus chinensis* seedlings. To test the hypotheses, we evaluated the effect of AMF on the growth, osmotic adjustment and ionic changes in seedlings of *Leymus chinensis* under the interaction of alkali and drought stresses.

## Materials and Methods

### Growth conditions

Seeds of *L. chinensis* were harvested from Songnen grassland in northeastern China (123°44′E, 44°44′N) in 2017 and employed in the present study. BGC HEB02 (https://www.uniprot.org/taxonomy/27381) was provided by the Institute of Plant Nutrition and Resources, Beijing Academy of Agriculture and Forestry Sciences, China.

A pot experiment was carried out in the greenhouse (relative humidity: 60 ± 5%; ambient temperature: 25 ± 1 °C; photoperiod: 16 h light (800 mmol m^−2^ s^−1^) followed by 8 h darkness) at Northeast Forestry University, Heilongjiang Province, China. The experiment was conducted under a completely randomized design with three factors: alkali stress (0, 100 and 200 mM NaHCO_3_), drought stress [0, 5 and 10% (w/v) PEG 6000] and mycorrhizal treatment (inoculated with AMF and noninoculated). Each treatment included four replicates, and one pot with eight seedlings was considered one replicate.

The filled seeds were selected and sterilized with 10% NaClO, rinsed twice with distilled water and germinated in petri dishes. After germination, the young seedlings were transferred to plastic pots (15 cm diameter) containing an autoclaved soil mixture (soil:sand 3:1, v:v). Each pot was inoculated with either 20 g of inoculant (a mixture of soil substrate, mycorrhizal root fragments, spores, and mycelia of https://www.uniprot.org/taxonomy/27381) for the inoculation treatment or 20 g of sterilized inoculant for the noninoculation treatment. We also added 30 mL of filtered (0.25-µm filter membrane) inoculant, which was free of mycorrhizal propagules, to the noninoculation treatment to maintain the same microorganism biota. The inoculants were added two cm below the seeds. One hundred and twenty days after sowing, the seedlings were treated with the corresponding treatment solutions as described above. The seedlings were harvested 7 days after the treatments were implemented.

### Seedling growth and AMF colonization measurements

The seedlings in each treatment group were collected and washed with distilled water. The separated shoots and roots were dried at 80 °C to constant weight, and the dry weight was finally measured. Thirty 1-cm fragments of fresh roots were used to measure mycorrhizal colonization. The fragments were stained with trypan blue (0.05%) following Phillips & Hayman (1970), and colonization was calculated by using the formula described by Lin et al. (2017): AM colonization (%) = (root length infected/root length observed) × 100.

### Na^+^ and K^+^ content measurements

A dry shoot sample (50 mg) was treated with 10 mL deionized water in a boiling water bath for 1 h. The extracts were used to measure Na^+^ and K^+^ contents by atomic absorption spectrometry (Z-5000, Hitachi, Japan) at 589 nm and 766.5 nm, respectively.

### MDA and proline content measurements

Thiobarbituric acid (0.6%. one mL) was used to extract malondialdehyde (MDA) from fresh shoots (200 mg), and the absorbance was then measured at 600, 532 and 450 nm (Wang et al., 2019a; Wang et al., 2019b Wang et al., 2019c). Proline was extracted with 3% sulfosalicylic acid for 1 h, and the absorbance was measured at 520 nm (Bates, Waldren & Teare, 1973).

### Statistical analysis

The data from four biological replicates were analyzed by using SPSS version 19.0 (Chicago, IL, USA). Analysis of variance (one-, two- or three-way ANOVA) followed by Tukey’s post-hoc test was used to determine the differences among each treatment group. The differences were considered significant at the 5% level.

## Results

### Root colonization

**Figure 1:**
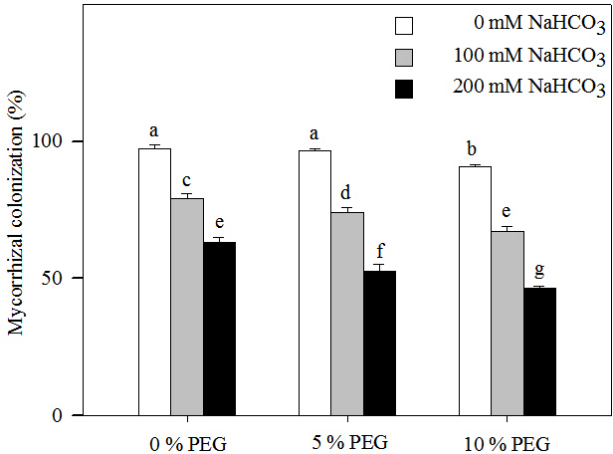
Mycorrhizal colonization rate in the seedlings of *Leymus chinensis* under alkalinity and drought stresses. The bars represent the means ± S.E. Different letters indicate a significant difference between the seedling with or without AMF.

Two-way ANOVA indicated that the mycorrhizal colonization of *Leymus chinensis* seedlings was affected by the alkali and drought treatment concentrations and the interaction of the two factors (Table 1). Mycorrhizal colonization decreased significantly with increasing alkalinity (Fig. 1). At the highest drought treatment concentration (10% PEG), the mycorrhizal colonization rate was 90.67% in 0 mM NaHCO_3_ but only 46.67% in 200 mM NaHCO_3_. Mycorrhizal colonization also significantly decreased with an increasing drought treatment concentration (Fig. 1). Under drought treatment, the mycorrhizal colonization rate was 97.33% in 0% PEG, 79.33% in 5% PEG and 63.33% in 10% PEG. Mycorrhizal colonization was decreased more markedly under the interaction of alkali and drought stresses than under alkali stress alone. For instance, mycorrhizal colonization in 200 mM NaHCO_3_ was reduced by 34% compared with that in the nonstress treatment but was decreased by 44% in the 10% PEG treatment.

**Table 1:** Three-way ANOVA of effects of inoculation (*In*), alkaliconcentration (*AC*) and drought concentration (*DC*), and their interactions on physiological parameter s in the seedlings of *Leymus chinensis*.

Dependent variable	Independent variable													
	*In*		*AC*		*DC*		*In*×*AC*		*In*×*DC*		*AC*×*DC*		*In*×*AC*×*DC*	
	*F* values	*P* values	*F* values	*P* values	*F* values	*P* values	*F* values	*P* values	*F* values	*P* values	*F* values	*P* values	*F* values	*P* values
Mycorrhizal colonization	–	–	481.09	0.00	40.36	0.00	–	–	–	–	3.24^*^	0.04	–	–
Dry weight	117.23	0.00	224.03	0.00	34.48	0.00	71.61	0.00	2.26	0.12	2.48^*^	0.06	1.92	0.13
Water content	48.98	0.00	408.23	0.00	128.62	0.00	21.83	0.00	1.71	0.2	3.63^**^	0.01	2.56	0.06
Na^+^	170.64	0.00	4993.62	0.00	37.54	0.00	51.53	0.00	4.39	0.02	10.91^***^	0.00	2.32	0.08
K^+^	36.29	0.00	1452.59	0.00	6.70	0.00	13.18	0.00	0.52	0.6	2.93^*^	0.03	2.06	0.11
Na^+^/K^+^	307.92	0.00	5248.03	0.00	61.10	0.00	78.69	0.00	3.63	0.04	15.33^***^	0.00	3.32	0.02
Proline	28.76	0.00	637.17	0.00	108.30	0.00	1.61	0.21	2.91	0.07	13.27^***^	0.00	8.16^*^	0.00
MDA	23.16	0.00	291.97	0.00	173.19	0.00	3.38	0.04	1.15	0.33	38.81^***^	0.00	0.34	0.85

**Notes.**

Date represent *F*-values at 0.05 level.

### Growth

**Figure 2:**
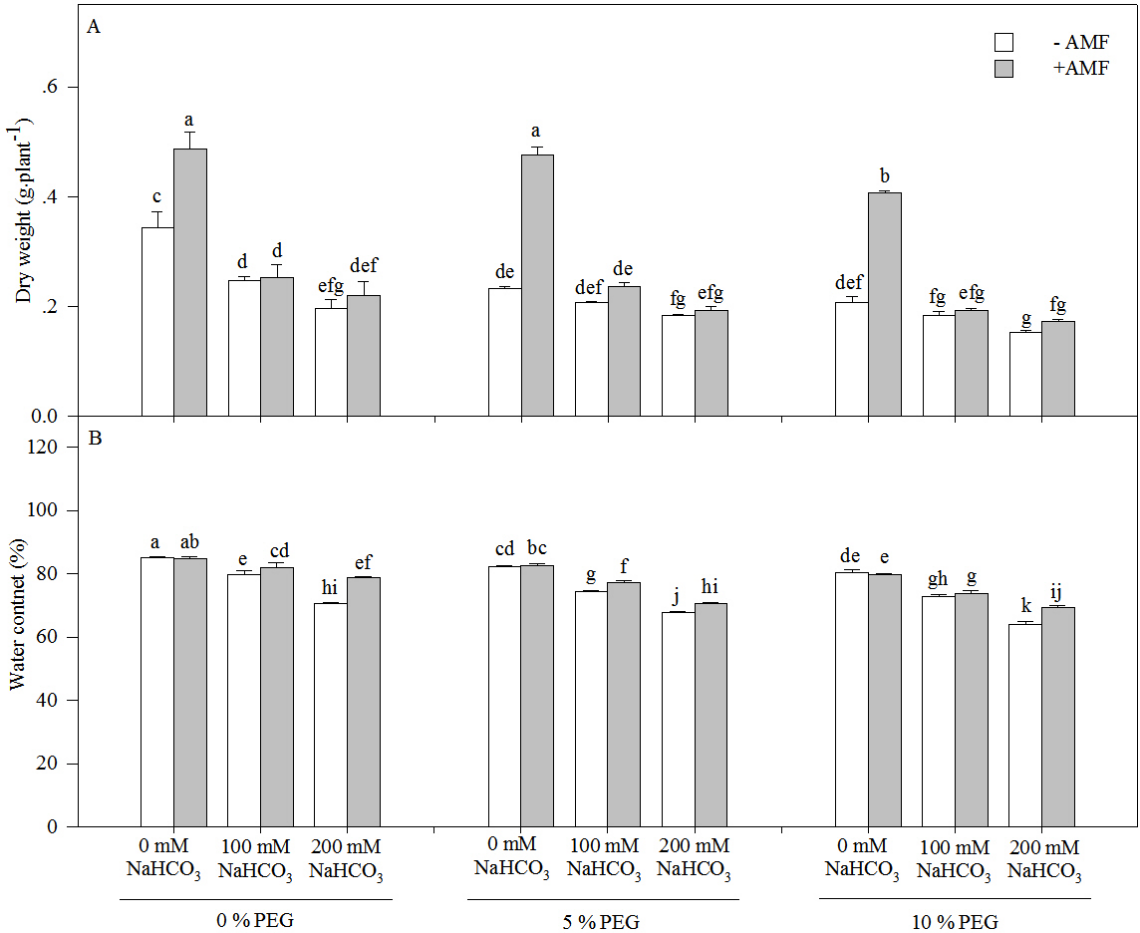
The dry weight (A) and water content (B) in the seedlings of *Leymus chinensis* under alkalinity and drought stresses. The bars represent the means ± S.E. Different letters indicate a significant difference between the seedlings with or without AMF under each treatments.

The dry weight and water content of the seedlings of *Leymus chinensis* significantly decreased with an increasing drought or alkali treatment concentration (Fig. 2 and Table 1). Moreover, the dry weight decreased more markedly under alkali stress alone than under the interaction of alkali and drought stresses. For instance, the dry weight in 200 mM NaHCO_3_ was reduced by 42.70% relative to the nonstress condition but was only decreased by 21.43% in 5% PEG and 25.83% in 10% PEG. However, AMF inoculation significantly increased the dry weight and water content of the seedlings of *Leymus chinensis* in each treatment. The dry weight of AM seedlings was improved by 41.77% in 0 mM NaHCO_3_, 2.68% in 100 mM NaHCO_3_ and 11.85% in 200 mM NaHCO_3_ compared with that of non-AM seedlings under alkali treatment. In addition, three-way ANOVA indicated that the dry weight and water content of *Leymus chinensis* seedlings were only affected by the interaction of inoculation and the alkali concentration (*In* × *AC*) and the interaction of the alkali concentration and the drought treatment concentration (*AC* × *DC*, Table 1).

### Na^+^ and K^+^ contents

**Figure 3:**
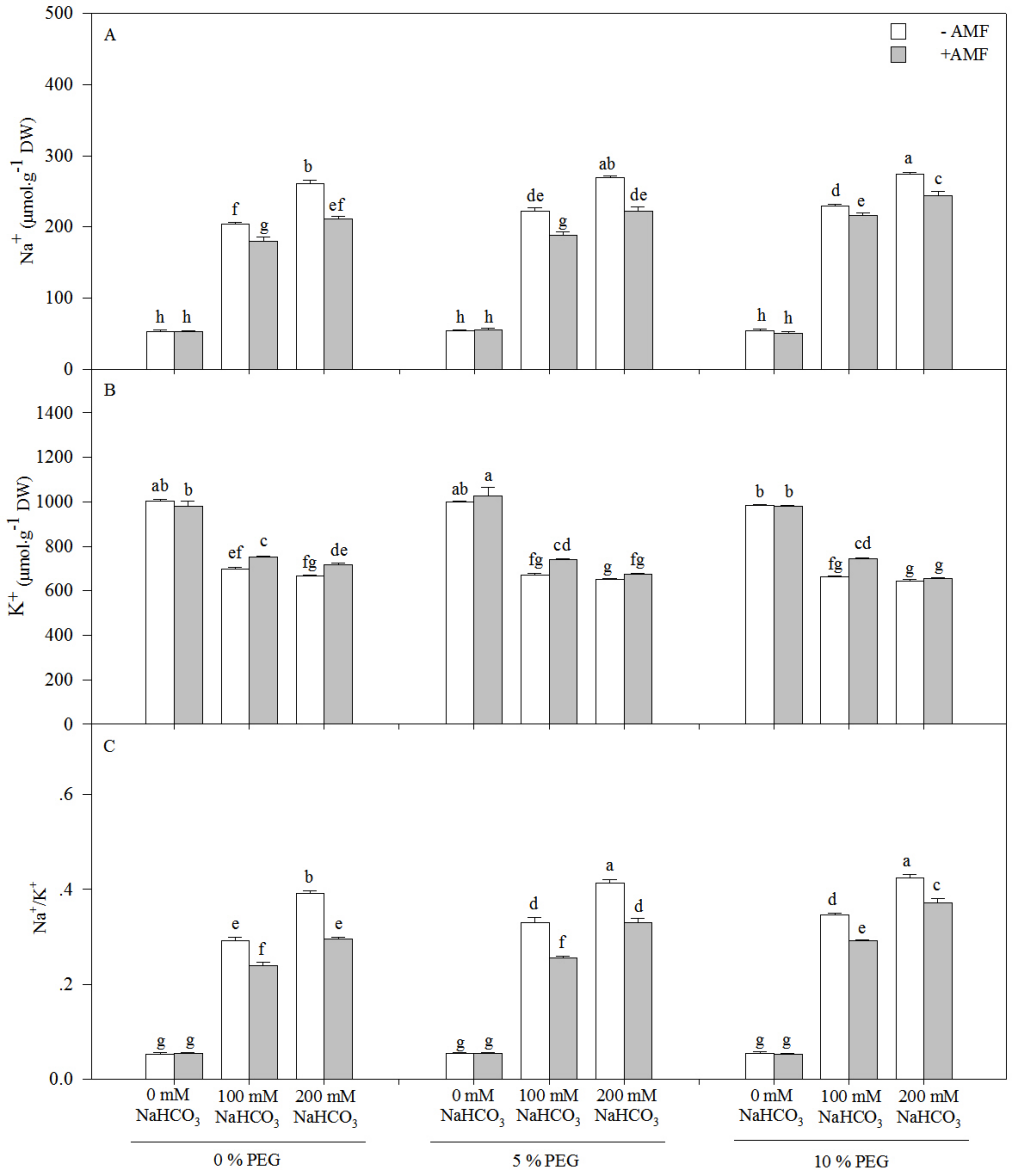
The contents of Na^+^ (A), K^+^ (B) and Na/K^+^ (C) in the seedlings *Leymus chinensis* under alkalinity and drought stresses. The bars represent the means ± S.E. Different letters indicate a significant difference between the seedlings with or without AMF under each treatments.

The Na^+^ content and Na^+^/K^+^ of the seedlings of *Leymus chinensis* increased significantly with an increasing alkali or drought treatment concentration, and AMF inoculation significantly reduced the Na^+^ content and Na^+^/K^+^, except under the 0 mM NaHCO_3_treatment with each drought treatment (Fig. 3 and Table 1). Compared with the non-AM seedlings, the Na^+^ concentration was reduced by 15.07% in the 100 mM NaHCO_3_ treatment and 17.50% in 200 mM NaHCO_3_ and 5% PEG treatment. However, the K^+^ content of the seedlings of *Leymus chinensis* decreased with an increasing alkali or drought treatment concentration. AMF inoculation increased the K^+^ content of the seedlings of *Leymus chinensis* under the alkali and drought treatments. Two-way ANOVA indicated that the interaction of inoculation and the alkali concentration (*In* × *AC*) and the interaction of the alkali concentration and the drought treatment concentration (*AC* × *DC*) significantly affected Na^+^, K^+^ and Na^+^/K^+^ in the seedlings of *Leymus chinensis*, while the interaction of inoculation and the drought treatment concentration (*In* × *DC*) only significantly affected Na^+^ and Na^+^/K^+^. Moreover, three-way ANOVA indicated that Na^+^/K^+^ was only affected by the interaction of the three factors (*In* × *AC* × *DC*) (Table 1).

### Lipid peroxidation and proline accumulation

**Figure 4:**
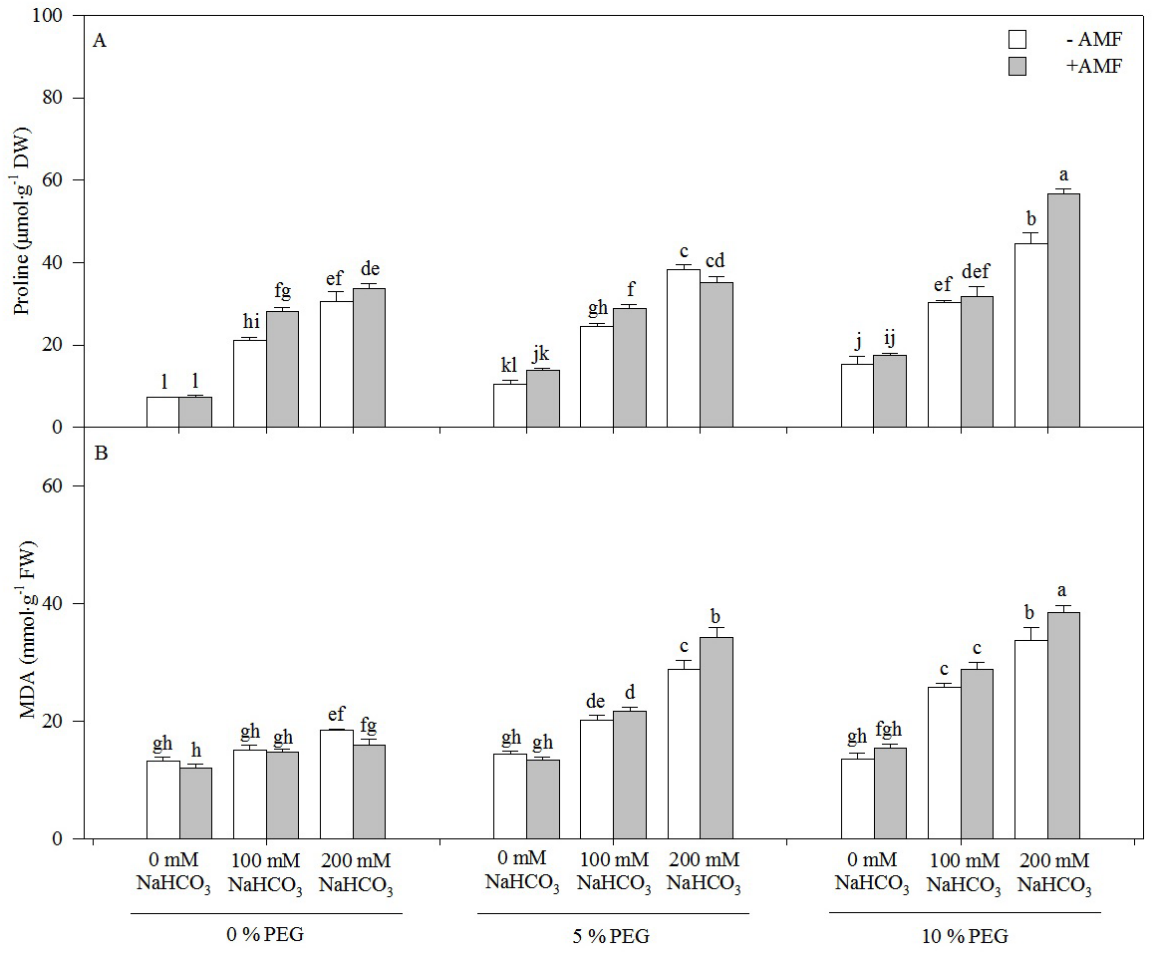
The contents of proline (A) and MDA (B) in the seedlings of *Leymus chinensis* under alkalinity and drought stresses. The bars represent the means ± S.E. Different letters indicate a significant difference between the seedlings with or without AMF under each treatments.

Both MDA and proline contents increased with an increasing alkali concentration in the seedlings of *Leymus chinensis*, but only the proline content increased with an increasing drought treatment concentration (Fig. 4). The proline content of the seedlings of *Leymus chinensis* was increased 1.83-fold in 100 mM NaHCO_3_ and 3.10-fold in 200 mM NaHCO_3_ relative to the 0 mM NaHCO_3_ treatment. Moreover, the interaction of alkali and drought stresses had a greater effect on MDA and proline than single alkali stress. The proline content in 200 mM NaHCO_3_ was increased 3.10-fold relative to the nonstress treatment, but it was only increased 2.66-fold in 5% PEG and 1.88-fold in 10% PEG. In contrast, the MDA content in 200 mM NaHCO_3_ was only increased 39.75% compared with that in the nonstress treatment, but it was increased 1.01-fold in 5% PEG and 1.48-fold in 10% PEG. AMF inoculation significantly increased the proline content of the seedlings of *Leymus chinensis* in each treatment except for 0 and 200 mM NaHCO_3_ in the 5% PEG treatment. However, the MDA content was reduced by AMF inoculation. Two-way ANOVA indicated that the interaction of inoculation and the drought treatment concentration (*In* × *DC*) significantly affected both MDA and proline contents in the seedlings of *Leymus chinensis*, while the interaction of inoculation and the alkali concentration (*In* × *AC*) only significantly affected MDA. In addition, three-way ANOVA indicated that the proline content of the *Leymus chinensis* seedlings was affected by the interaction of the three factors (*In* × *AC* × *DC*) (Table 1).

## Discussion

**Figure 5:**
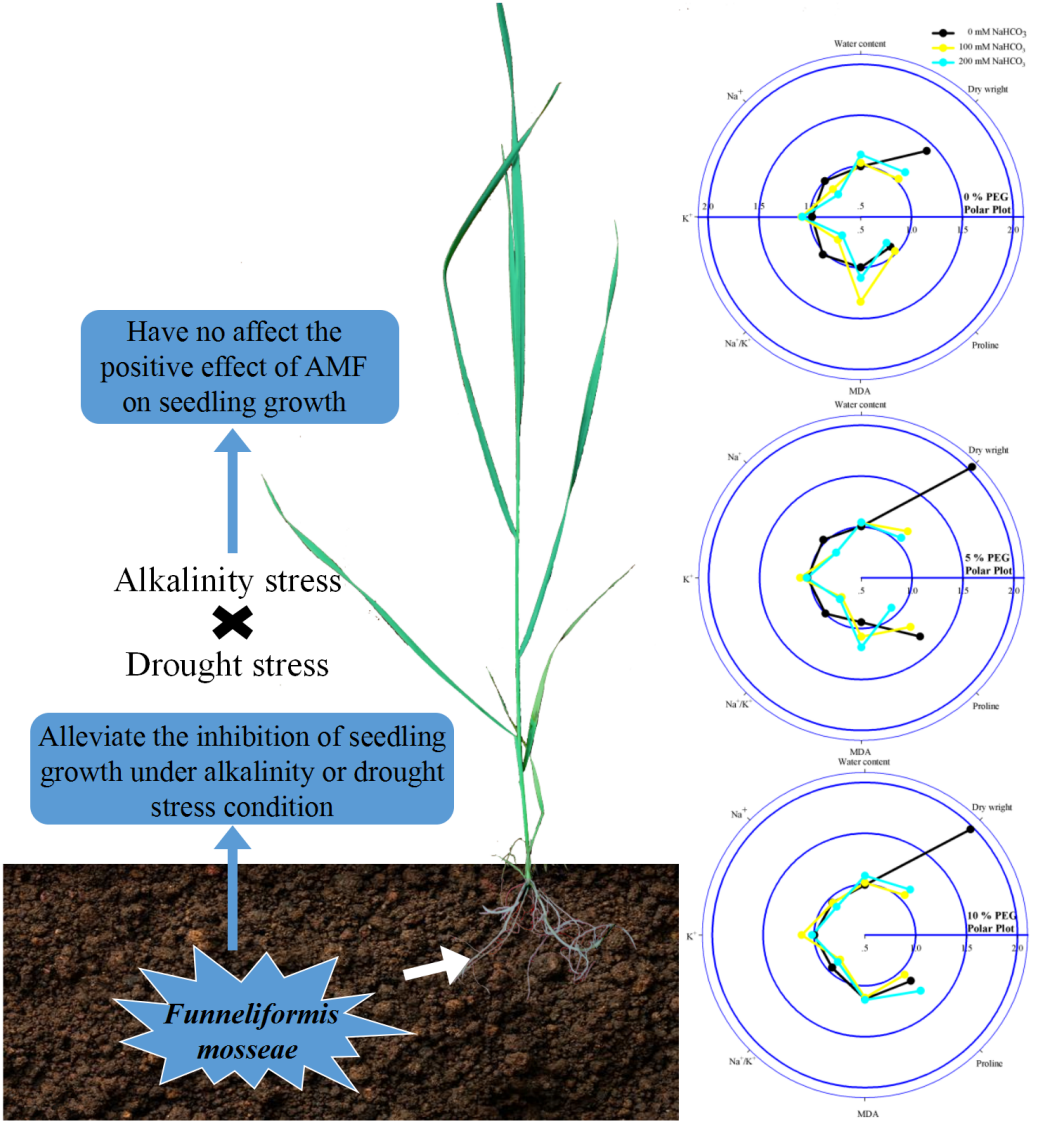
Diagram on the left illustrating the combined effect of alkalinity and drought stress in the seedlings of *Leymus chinensis* with AMF. The radar plots on the right show the effect of AMF colonization on the parameters in 0, 100 and 200 mM NaHCO_3_ with 0, 5 and 10% PEG. All data were normalized to the reference non-AMF seedling.

Alkali and drought are severe environmental stresses that inhibit both plant growth and soil microorganism development (Liu et al., 2014). In this study, AMF colonization decreased with increasing NaHCO_3_ or PEG levels and was much greater under NaHCO_3_ treatment than under PEG treatment (Fig. 5). The results indicated that alkali and drought stress both inhibited the development of AMF and that the inhibitory effect of alkali stress was much stronger than that of drought stress. Similar results have been obtained in *Puccinellia tenuiflora* infected by *R. intraradices* (Yang et al., 2020a; Yang et al., 2020b), *Knautia arvensis* infected by *Glomus* sp. (Doubková, Vlasáková & Sudová, 2013) and *Capsicum annuum* infected by *Rhizophagus irregularis* (Pischl & Barber, 2017). The reduction of AMF development is due to the inability of spores to germinate or the inhibition of host root development, preventing the establishment of a sufficient symbiotic relationship (Lenoir, Fontaine & Lounès-Hadj, 2016). Moreover, the interaction of alkali and drought stresses decreased AMF colonization, and the inhibitory effect was much stronger than that of either alkali or drought stress alone. The results indicated that the interaction of alkali and drought stresses had a stronger inhibitory effect on the development of AMF. However, the study also showed that the roots of *Leymus chinensis* formed good symbiotic relationships with AMF under the interaction between alkali and drought stresses.

A previous study reported that alkali stress affected nutrient uptake, ion homeostasis, the organic acid balance and especially pH stability in plant cells due to the combined effects of high-pH stress, osmotic stress, and ion toxicity and ultimately strongly inhibited plant growth (Zhao et al., 2016). Jia et al. (2018) reported that soil drought also inhibited plant growth by affecting nutrient uptake, restricting transpiration rates, and damaging active transport and membrane permeability. In accord with previous studies, we found that *Leymus chinensis* seedling growth was inhibited under alkali or drought stress. In addition, we found that seedlings of this species inoculated with AMF showed a higher water content and dry weight than seedlings without AMF under alkali or drought stress, indicating that mycorrhizal colonization relieved growth inhibition and improved water conditions in seedlings of *Leymus chinensis* (Fig. 5). AMF can form extensive hyphal networks in host roots, which extend the length of the host roots, allowing them to absorb more mineral nutrients and water (Lenoir, Fontaine & Lounès-Hadj, 2016; Pepe, Giovannetti & Sbrana, 2016). This is the main reason that the growth of the seedlings was improved by mycorrhizal colonization under alkali and drought stresses. Moreover, the dry weights and water contents of *Leymus chinensis* seedlings were greatly decreased under the interaction of alkali and drought stresses, indicating that the interactive effect of alkali and drought stresses caused more inhibition of plant growth than single alkali or drought stress. However, an no interactive inhibitory effect was found in the seedlings of *Leymus chinensis* colonized by AMF.

In addition to osmotic stress and nutrient deficiency caused by drought and alkali stresses, soil alkalinity also causes ionic damage due to the accumulation of Na^+^ and inhibition of K^+^ in plants; because Na^+^ competes with K^+^ for major binding sites in key metabolic processes in the cytoplasm, such as enzymatic reactions, protein synthesis and ribosome functions, these changes induce physiological disorders such as photosynthesis inhibition Flowers, Hajibagherp & Yeo, 1991), stomatal closure (Niu et al., 2018) and leaf cell dehydration (Li et al., 2010). The homeostasis of intracellular ion concentrations is critical for the metabolism of living cells. Plant cells have to keep the concentrations of toxic ions low and accumulate essential ions through the proper regulation of ion flux (Yan et al., 2013). In the present study, alkali stress induced the accumulation of Na^+^ and the depletion of K^+^ in seedlings of *Leymus chinensis*. In addition, we found that AMF inoculation reduced the Na^+^ content and increased the K^+^ content of the seedlings of *Leymus chinensis*, which maintained a lower Na^+^/K^+^ in the AMF seedlings, indicating restricted translocation of Na^+^ from roots to shoots in *Leymus chinensis* seedlings colonized by AMF (Fig. 5). There are two possible explanations for this phenomenon. First, AMF-inoculated plants could retain Na^+^ inside intraradical fungal hyphae and root cell vacuoles to restrict the transfer of this toxic ion from roots to shoots (Parvin et al., 2020). Second, AMF symbiosis might trigger the expression of Na^+^/H^+^ transporters that allow plants to sequester Na^+^ in vacuoles and limit the entry of Na^+^ into plant root systems (Ruiz-Lozano, Porcel & Gomez, 1995). Moreover, the Na^+^ content and Na^+^/K^+^ of the seedlings of *Leymus chinensis* were increased more markedly under the interaction of alkali and drought stresses than under alkali or drought stress alone, which reflected severe ionic toxicity under the interaction of these stresses in the seedlings of *Leymus chinensis*. Furthermore, the positive effect of AMF colonization was decreased under the interaction of alkali and drought stresses compared with that under alkali stress alone, which restricted the translocation of Na^+^ from roots to shoots. This was consistent with the finding that the development of AMF declined to a greater extent under the interaction of alkali and drought stresses in the present study.

In general, the MDA content is used to evaluate lipid peroxidation levels. The accumulation of MDA is always due to increases in reactive oxygen species (ROS), indicating oxidative damage to plants (Bistgani et al., 2017; Mohasseli, Farbood & Moradi, 2020). Here, alkali stress increased the lipid peroxidation level in the seedlings of *Leymus chinensis*, indicating oxidative damage in the seedlings of this species under alkali stress (Fig. 5). Moreover, we found that the MDA content of the seedlings did not change under drought stress. The results indicated that the degree of drought stress recorded in this study was relatively low for *Leymus chinensis* and did not cause severe oxidative damage. In addition, we found that AMF inoculation reduced the MDA content, which indicated that AMF colonization alleviated the oxidative damage caused by alkali stress in *Leymus chinensis*. Moreover, the MDA content of the seedlings was much higher under the interaction of alkali and drought stresses than under alkali or drought stress alone, indicating that the seedlings suffered more severe oxidative damage under the interaction of the two stresses. However, the alleviating effect of AMF colonization did not change under the interaction of alkali and drought stresses.

Proline is considered a low-molecular-weight osmolyte that is involved in osmotic adjustment and scavenges ROS in plants under abiotic stresses (Chun, Paramasivan & Chandrasekaran, 2018). Plant species exhibiting higher accumulation of osmolytes show improved tolerance and growth performance under stress through the maintenance of tissue water contents and protein structure and function (Begum et al., 2019). In this study, the accumulation of proline in the seedlings of *Leymus chinensis* indicated that under alkali or drought stress, the seedlings of this species could accumulate proline as an osmotic regulator to maintain osmotic balance in the protoplasm (Fig. 5). In addition, we found that AMF inoculation improved the proline content of the seedlings under alkali or drought conditions. A previous study showed that proline acts not only as an osmotic regulator but also as a nitrogen and carbon source for AMF (Keller & Hohn, 1997). Hence, the increase in proline in the AMF-colonized seedlings indicated a better osmotic regulation ability than in the seedlings without AMF and a mutualistic symbiotic relationship of *Leymus chinensis* and https://www.uniprot.org/taxonomy/27381. Moreover, the osmotic stress experienced by the *Leymus chinensis* seedlings was more severe under the interaction of alkali and drought stresses than under either alkali or drought stress alone, which was reflected in the higher proline content under the alkali-drought stress interaction. A similar tendency of proline was found in the *Leymus chinensis* seedlings colonized by AMF.

## Conclusion

The application of either alkali or drought stress inhibited the seedling growth of *Leymus chinensis*. The interaction of alkali and drought stresses had a stronger inhibitory effect on seedling growth in this species than either alkali or drought stress alone. Mycorrhizae could alleviate the inhibitory effect on seedling growth under alkali or drought stress conditions. The interaction of alkali and drought stresses did not affect the alleviating effect of arbuscular mycorrhizal fungi on seedling growth. The fungi only improved the osmotic regulation ability and ionic balance in *Leymus chinensis*. These findings provide an improved understanding of the effects of alkali and drought stresses and their interaction on AM plants.

## Supplemental Information

10.7717/peerj.12890/supp-1 Data S1 Raw data Infection rate, dry weight, water content, proline, MDA and potassium-sodium ions content.

## Additional Information and Declarations

### Data Availability

The following information was supplied regarding data availability: The raw measurements are available in the Supplementary File.
